# Generative AI in medicine: use and acceptance among professionals in orthopedics and traumatology: a survey-based analysis in Germany

**DOI:** 10.1038/s41598-026-70337-4

**Published:** 2026-09-09

**Authors:** Philipp Zehnder, Frederik Aasen-Hartz, Michael Zyskowski, Conrad Ketzer, Tobias Resch, AG Digitalisierung der DGOU, Peter Biberthaler, Dominik Pförringer

**Affiliations:** 1 Department of Trauma Surgery, Klinikum rechts der Isar, TUM University Clinic, Technical University Munich, Ismaningerstr. 22, 81675 Munich, Germany; 2 Digitalization Working Group, German Society of Orthopaedics and Traumatology, Berlin, Germany

**Keywords:** AI in scientific writing, ChatGPT, Digital health tools, Ethics in AI use, Generative AI, medical publishing, Health care, Mathematics and computing, Medical research

## Abstract

Generative AI tools such as ChatGPT (OpenAI, San Francisco, CA, USA) are showing remarkable capabilities in generating qualitative text, displaying potential to revolutionize daily life and professional practices, including medical diagnostics and education. The use of ChatGPT in publishing, especially its legal and ethical implications, is subject of growing scientific interest and discussion. The underlying study aims to understand the current status, usage patterns, and future expectations of generative AI among healthcare professionals. An anonymous online survey among healthcare professionals was distributed to members of the German society for orthopedics and traumatology (February and March 2024). The questionnaire comprised 12 questions regarding individual use of and attitude towards artificial intelligence. The responses of two age-dependent groups (group 1 ≤ 45 years vs. group 2 > 45 years) were compared. A total of 375 of 504 invited participants completed the survey (response rate 74%); 177 respondents were aged ≤ 45 years. Awareness of ChatGPT was high in both groups, while prior self-reported use was more common in group 1 (73% vs. 62%, *p* = 0.03). Respondents aged ≤ 45 years more often reported that they could imagine using ChatGPT for manuscript-related tasks (59% vs. 44%, *p* = 0.001), whereas respondents aged > 45 years more frequently supported disclosure of ChatGPT use in manuscript preparation (86% vs. 77%, *p* = 0.001). The age groups differed significantly in professional role distribution. In an adjusted analysis including professional position, the association between age group and AI use remained similar but not significant (unadjusted OR 1.6, 95% CI 1.1–2.4; adjusted OR 1.6, 95% CI 1.0–2.6; p 0.053). In this cohort of DGOU members, awareness of generative AI was high and self-reported use was common. Younger respondents reported more frequent use of ChatGPT and related digital tools. However, these findings should be interpreted cautiously because they reflect self-reported cross-sectional data from one professional society and do not demonstrate actual integration of AI into clinical or academic workflows. The creation of an ethical framework is therefore essential and our participants want the use to be clearly identified.

**Supplementary Information:**

The online version contains supplementary material available at https://doi.org/10.1038/s41598-026-70337-4.

## Introduction

The rapid advancement of artificial intelligence (AI) has stimulated extensive discussion regarding its impact on creative, academic, and clinical processes. Among the most prominent generative AI systems is ChatGPT (OpenAI, San Francisco, CA, USA), which has attracted considerable attention, particularly in relation to its implications for scientific practice^1,2^. A PubMed ^®^ search has yielded a total of 4625 results for the keyword “ChatGPT” and a total of 2603 results for the keywords “generative AI”, 1022 in 2023 alone (Status April 01st 2024). The relevance of generative AI in medicine is further reinforced by the widespread use of online resources for health-related information^3^. A large proportion of internet users search for medical information online before or alongside professional healthcare consultation^4^. Consequently, the quality, readability, and reliability of digital health information may directly influence patients’ understanding of diseases, expectations toward treatment, and participation in shared decision-making^5^. This is particularly relevant in view of health-literacy recommendations, which emphasize that patient-directed educational materials should be written in a way that is understandable to a broad audience^6^. This underlines the increasing importance and relevance of generative AI. In particular, the advanced AI language model ChatGPT has shown remarkable capabilities in generating human-like text, which has sparked interest and discussions about its potential to revolutionize daily lives. However, the potential is not only seen in our everyday tasks, but also in a medical context. Numerous publications are currently being produced dealing with AI as a support in diagnostics^7–10^. This involves the analysis of radiological images or the evaluation of pathological samples. Another major branch seems to revolve around the use of ChatGPT for educational purposes^11–13^. Realistic medical cases can be simulated and even the questions for subsequent exams can be created^14,15^. Not only publishing via ChatGPT and its various applications is becoming increasingly popular. Publishing with the help of ChatGPT is also the subject of scientific discussion^16,17^. Scientific discussion has increasingly focused on the potential role of generative AI in manuscript preparation, literature-related tasks, and academic writing support, as well as on the associated ethical, legal, and transparency-related challenges^18–20^. With this study, we aimed to explore awareness, self-reported use, and attitudes toward generative AI among medical professionals.

Therefore, an anonymous survey among healthcare professionals was distributed online, it aimed at evaluating the following research questions:

### RQ1

To what extent have physicians adopted generative AI in their professional practice, and what are their attitudes toward it?

### RQ2

In what specific ways do physicians utilize generative AI tools?

### RQ3

Which future expectations do physicians have from generative AI solutions?

## Methods

This study has been approved by the ethics committee of the medical faculty of Technical University Munich (TUM), Germany (Project number: 2024-57-S-CB).

The research protocol and the survey questions were developed (P.Z., D.P.) based on the ongoing work of D.P. for the committee for Digitalization of the DGOU (German society for orthopedics and traumatology). Prior to distribution, the survey was subjected to an internal review within the department to evaluate the clarity, relevance, and comprehensibility of the questions. No formal pilot testing, validity assessment, or reliability testing was performed. An anonymous online survey among healthcare professionals was sent by e-mail to members of the DGOU. The evasys (evasys GmbH, Lüneburg, Germany) platform was used to create the questionnaires (see original questionnaire in supplementary material). Participants were asked to complete the survey within 60 days (February and March 2024). The questionnaire comprised 12 questions and took about 5 min to complete. The utilization of artificial intelligence, its acceptance, and the usage patterns of digital tools in the context of medical manuscript writing was assessed. The Data were organized in Excel (Version 2019, Microsoft, Redmond, USA) and two groups were formed: Participants up to the age of 45 (group 1) and participants older than 45 (group 2). The cut-off of 45 years was chosen a priori to approximately distinguish between earlier or mid-career participants and those at a more advanced career stage within the clinical hierarchy. This threshold was intended to provide clinically and professionally interpretable groups rather than to imply a biologically defined age boundary.

Statistical analysis was conducted using SPSS (Version 27.0, IBM Corp., Armonk, USA). The Kolmogorov-Smirnov test was used to assess for normal distribution. If normal distribution was confirmed, students’ t-test was used to compare differences in means. Otherwise, Mann-Whitney U test was used for analysis. Categorical variables were compared using the Fisher exact test and the chi-square test (Pearson chi-square). To explore potential confounding by professional position, an additional binary logistic regression model was fitted. The dependent variable was prior self-reported use of ChatGPT, coded as yes versus no. Age group (≤ 45 years vs. >45 years) was included as the main independent variable, and professional position was included as an adjustment variable. *P* < 0.05 was considered statistically significant. Because several questionnaire items were compared between age groups, these analyses should be interpreted as exploratory. No formal adjustment for multiple testing was performed.

## Results

375 of 504 participants completed the questionnaire (response rate 74%). 177 participants (47%) were aged 45 or younger and were assigned to group 1, the other 198 (53%) formed group 2. Regarding the clinical role of the participating physicians a significant difference could be detected. There were significantly more residents (35% vs. 1%) and significantly fewer chief physicians (4% vs. 37%) in group 1 than in group 2 (*p* = 0.001). Both groups were similarly familiar with ChatGPT, although group 1 had already used the program more frequently (73% vs. 62%, *p* = 0.03). If the participants use ChatGPT, they have done so for similar tasks. In both groups, ChatGPT was most frequently used for broad text-related tasks, including summarizing texts (without further specification of text type; 41% vs. 33%), writing emails (38% vs. 28%), and translation (32% vs. 24%). In group 1, significantly more participants could imagine using the program (59% vs. 44%, *p* = 0.001) or have already used it (19% vs. 17%, *p* = 0.001) for writing a scientific manuscript. A significantly lower proportion in this group categorically rules out using the program (22% vs. 40%, *p* = 0.01, Table 1).

**Fig. 1:**
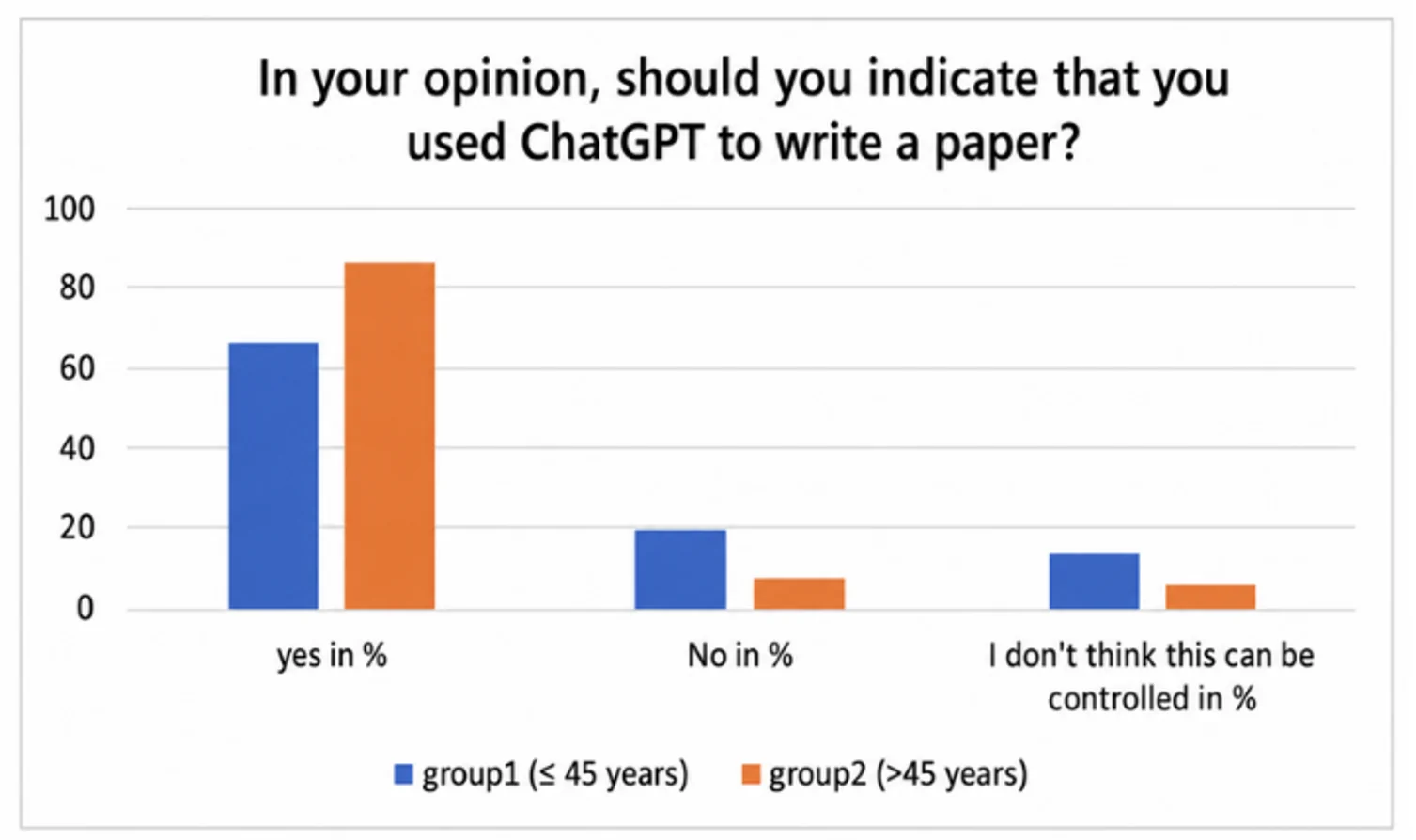
Should you indicate that you used ChatGPT to write a paper? (responses in %, *p* = 0.001)

In the younger age group, significantly fewer participants stated that the use of ChatGPT for creating a paper should be disclosed (77% vs. 86%, *p* = 0.001) and the opinion that this could not be controlled anyway was more widely shared (14% vs. 6%, *p* = 0.001, Fig. 1).

**Table 1:** Demographic characteristics and reported AI use across the two age groups (responses in %).

	Group 1 (≤ 45 years (*n* = 177))	Group 2 (> 45 years (*n* = 198))	*p*-value
Please indicate the function in your clinic?			
Resident	34.7%	1.0%	0.001
Specialist	15.9%	12.2%	
Senior physician	35.8%	27.0%	
Chief physician	4.0%	36.7%	
Other	9.7%	23.0%	
Do you know ChatGPT?	97.7%	94.4%	0.102
Have you already used ChatGPT?	73.1%	61.9%	0.028
For what purpose have you already used ChatGPT (multiple answers possible)?			
To summarize a text	41.2%	32.8%	0.092
To write an e-mail	37.9%	27.8%	0.038
To create a presentation	18.1%	15.5%	0.446
As a translation tool	32.2%	23.7%	0.068
To draw pictures (DALL-E)	13.0%	11.6%	0.685
For analyzing data	6.8%	3.5%	0.153
For writing a paper	18.1%	12.1%	0.106
To search for sources	20.9%	20.7%	0.963
Could you imagine using ChatGPT to write a paper or have you already used it?			
I could imagine using it	58.9%	43.6%	0.001
I have already used it	19.4%	16.9%	
I don’t think I will use the program	21.7%	39.5%	

When asked which aspects they considered central to scientific authorship when writing a paper, significant differences between age groups emerged in three areas. In Group 1, data analysis was considered more important (88% vs. 79%, *p* = 0.024). However, writing the manuscript (44% vs. 58%, *p* = 0.009) and finding relevant sources (42% vs. 53%, *p* = 0.049) were seen as less important. Participants in group 1 reported more frequent use of conventional search engines such as Google (81% vs. 68%, *p* = 0.004), digital translation programs (50% vs. 36%, *p* = 0.005), citation assistance programs (83% vs. 51%, *p* = 0.001), and tools for statistical analysis (92% vs. 79%, *p* = 0.001). The hopes and expectations for AI are largely similar in both groups. Both express a desire for assistance in finding relevant sources (80% vs. 76%, *p* = 0.298), a tool for detecting plagiarism (62% vs. 64%, *p* = 0.681), and a smart news ticker to identify current topics (53% vs. 54%, *p* = 0.847, Table 2). The association between age group and AI use remained similar after adjustment for professional position (unadjusted OR 1.6, 95% CI 1.1–2.4; adjusted OR 1.6, 95% CI 1.0-2.6), suggesting little evidence of confounding by position. However, the adjusted association did not reach conventional statistical significance (*p* = 0.053), indicating limited precision.

**Table 2:** Differences between the groups regarding perceived key aspects of scientific authorship, use of digital tools, and expectations toward generative AI (responses in %).

	Group 1 (≤ 45 years (*n* = 177))	Group 2 (> 45 years (*n* = 198))	*p*-value
In your opinion, what is the main focus of a scientific achievement when writing a paper? (multiple answers possible)			
The idea for the paper	80.2%	73.2%	0.11
The collection of data	63.3%	60.6%	0.595
When analyzing the data	87.6%	78.8%	0.024
Writing the paper	44.1%	57.6%	0.009
When searching for and citing qualified literature (PubMed)	42.4%	52.5%	0.049
Which of the following digital tools do you use when writing a paper? (multiple answers possible)			
Search engine (Google etc.)	81.4%	68.2%	0.004
Meta-database (PubMed, Scopus etc.)	94.4%	89.9%	0.113
Digital translation programs (DeepL, iTranslate etc.)	50.3%	35.9%	0.005
Citation assistance programs (endnote, citavi etc.)	83.1%	51.2%	0.001
Tools for statistical analysis (SPSS, Excel, etc.)	92.1%	79.3%	0.001
Other	14.1%	14.6%	0.886
What would you hope or wish for from a generative AI like ChatGPT in the future? (multiple answers possible)			
Help with the search for relevant sources	80.2%	75.8%	0.298
Complete formulation of manuscripts	33.3%	24.2%	0.052
Detection and analysis of possible plagiarism	61.6%	63.6%	0.681
Search function for ongoing tracking of current topics, aka smart news ticker	52.5%	53.5%	0.847
Other features	6.8%	8.6%	0.513

## Discussion

This survey provides a cross-sectional overview of awareness, self-reported use, and attitudes toward generative AI among members of the German Society for Orthopedics and Traumatology. Awareness of ChatGPT was high in both age groups, and self-reported prior use was common. Respondents aged ≤ 45 years reported more frequent use of ChatGPT and other digital tools, and they were more likely to consider using ChatGPT for manuscript-related tasks. By contrast, respondents aged > 45 years more often favored explicit disclosure of ChatGPT use in manuscript preparation.

These findings should be interpreted cautiously. Although age-group differences were observed, the two groups also differed substantially in professional role, with more residents in the younger group and more chief physicians in the older group. This raised the possibility that the observed differences might reflect career stage rather than age alone. To address this issue, we performed an adjusted analysis including both age group and professional position. In this model, the estimated association between younger age and self-reported AI use remained similar in magnitude. However, the adjusted association did not reach conventional statistical significance, indicating limited precision. Thus, while the analysis did not provide strong evidence that professional position materially explained the observed age-group difference, residual confounding by career stage cannot be excluded.

An important observation of this study is that self-reported use of generative AI was already relatively common, whereas the idea of fully delegating manuscript writing to AI was viewed more cautiously. Respondents most often reported using ChatGPT for tasks such as summarizing text, writing emails, and translation, whereas complete manuscript formulation was less frequently endorsed as a desirable future function. This pattern suggests that, within this cohort, generative AI is currently perceived more as a supportive tool for selected tasks than as a substitute for the full scientific writing process. These findings should also be interpreted within the broader context of online health-information seeking. Digital platforms, including websites, search engines, social media channels, and video-based platforms such as YouTube, remain widely used sources of medical information for both patients and healthcare professionals. This relevance became particularly apparent during the COVID-19 pandemic, when rapidly evolving diagnostic and treatment recommendations led many users to seek information online. However, the quality, reliability, and readability of online health information may vary substantially across platforms, medical topics, and languages. Studies evaluating non-English patient education materials have shown that readability and accessibility concerns are not limited to English-language content^21^. This underlines the importance of health literacy and of presenting medical information in a format that can be understood by a broad audience.

Another relevant finding concerns transparency in scientific writing. Most respondents agreed that the use of ChatGPT in manuscript preparation should be disclosed, although this view was more common among older participants. This result is noteworthy because it indicates broad support for transparency even in a cohort with high awareness and substantial self-reported use of generative AI. At the same time, a proportion of respondents, particularly in the younger group, considered disclosure difficult to enforce^22,23^. Looking at the general use of digital tools when writing a paper, the younger participants in the study also use the other existing programs significantly more often. Two studies from 2008 to 2009 also show how younger people were much more open towards using digital services like the internet^24,25^. Nowadays, the use of the internet is rarely debated, and it seems possible that ChatGPT could follow a similar trajectory in the coming years. Initially, the medical community was also skeptical about the digital search platform PubMed^®^ for example. Today, most of medical research can be found at PubMed^®^ and almost all of the participants in our cohort use the platform^26^. But how can ChatGPT support medical professionals right now? The creation of a finished scientific manuscript still seems to pose a problem at the moment^27^. However, charts and graphs can already be produced relatively reliably. This reflects exactly the desire of our participants for support from ChatGPT when composing a manuscript, but not for independent implementation. However, a key desire among our participants was assistance in identifying relevant literature. However, this still seems to pose a challenge for generative AI. In the present study from May 2024, it was shown that the use of ChatGPT to search for useful literature references is inaccurate and difficult to implement. Perhaps this will improve soon. For example, there are plans to adapt the creativity in favor of coherence with a special temperature controller^28^. Many of our participants may be skeptical about using AI, or at least about admitting to do so, as this must be explicitly stated when submitting a paper^29^. However, it is not yet known to what extent this will lead to an adaptation of the review process by the journals. One therefore has the impression that many of publishing authors are critical of the programs. Nevertheless, the total number of users and probably also the number of authors using the program for such purposes is increasing^30^. The constant improvement of the programs suggests that it may soon be possible to conduct better literature search or even prepare entire manuscripts^31^. This will also fuel both the general as well as specifically the ethical debate on the subject. So far, only the academic challenges with regard to the accuracy of the data generated by AI or the naming of ChatGPT as a co-author have been mentioned^32^. What we also need, however, are resolutions from the medical community that define the handling of AI in connection with the publication^33^. We can contribute here with this survey and show that AI is already being used to create medical manuscripts. However, there is a clear vote in favor of making its use recognizable and thus reliably identifying potential plagiarism.

## Conclusions

In this survey of members of the German Society of Orthopedics and Traumatology, awareness of generative AI tools was high and self-reported use was common in both age groups. Respondents aged ≤ 45 years reported more frequent use of ChatGPT and other digital tools, as well as greater willingness to consider ChatGPT for manuscript-related tasks. Overall, generative AI was perceived primarily as a supportive tool for selected academic and administrative tasks rather than as a substitute for professional expertise or independent scientific work.

These findings should be interpreted cautiously, as they reflect self-reported behavior within one professional society and may be influenced by differences in professional role and career stage between age groups. The study therefore provides a cross-sectional snapshot of attitudes and reported use rather than evidence of actual integration of generative AI into clinical or academic workflows.

While generative AI may facilitate access to information and support selected educational, administrative, or literature-related tasks, its limitations must be clearly recognized. AI-generated information should not be used as a primary source of patient information or as an independent basis for clinical decision-making. In particular, risk-benefit assessment, individualized treatment recommendations, and recognition of warning signs require professional oversight, clinical expertise, and guideline-based judgment.

### Limitations

This study has several limitations. First, the survey was conducted among members of a single professional society, which may limit generalizability and introduces the possibility of selection bias. Second, although the response rate was high, no information was available on non-responders; therefore, non-response bias cannot be excluded. Third, the study relied entirely on self-reported behavior and attitudes, and actual use of generative AI tools was not independently verified. Fourth, responses may have been influenced by social desirability bias, particularly regarding attitudes toward transparency and disclosure of AI use in manuscript preparation.

Fifth, because the two age groups differed substantially in professional role, confounding by career stage had to be considered when interpreting age-related differences. To address this, we performed an adjusted analysis including both age group and professional position. In this analysis, the estimated association between age group and self-reported AI use remained essentially unchanged, suggesting that professional position did not materially confound this association in our cohort. However, because age and career stage are closely related and only limited adjustment was possible, residual confounding cannot be fully excluded.

Sixth, the questionnaire was developed for the purpose of this survey and was not subjected to formal pilot testing, psychometric validation, or reliability assessment. Therefore, the findings should be interpreted as exploratory and descriptive.

Finally, due to its cross-sectional design, this study provides only a snapshot of self-reported behavior and attitudes and does not allow conclusions about causal relationships or temporal trends. In addition, the questionnaire was designed to provide a broad descriptive overview of awareness, self-reported use, and attitudes toward generative AI rather than a detailed assessment of specific clinical or academic workflows. Consequently, the study did not evaluate the use of generative AI in patient education, documentation, care pathways, clinical decision support, or productivity outcomes in detail.

## Supplementary Information

Below is the link to the electronic supplementary material.

Supplementary Material 1

## Data Availability

The data approved by the Ethics Committee of the Technical University of Munich that support the findings of this study are subject to access restrictions and are therefore not publicly available. However, the data can be obtained upon reasonable request and with the permission of the Ethics Committee of the Technical University of Munich.
