# Supplementary material for: Generative AI in medicine: use and acceptance among professionals in orthopedics and traumatology: a survey-based analysis in Germany
Source: Sci Rep. 2026 Sep 9;16:28239. doi: 10.1038/s41598-026-70337-4 (PMC13558731; doi:10.1038/s41598-026-70337-4)
Supplement: Supplementary file 1 [file 41598_2026_70337_MOESM1_ESM.docx]

## **Questionnaire** Generative AI in medicine

**1. Please indicate your gender**

Ο female
Ο male
Ο diverse

**2. Please assign yourself to an age group**

Ο < 30 years
Ο 30–45 years
Ο 46–60 years
Ο > 60 years

**3. Please indicate your position in the hospital**

Ο Resident physician
Ο Specialist physician
Ο Senior physician
Ο Head of department
Ο Other

**4. Are you familiar with ChatGPT?**

Ο Yes
Ο No
Ο No answer

**5. Have you used ChatGPT before?**

Ο Yes
Ο No
Ο No answer

**6. For what purposes have you used ChatGPT? (Multiple answers possible)**

Ο To summarize a text
Ο To write an email
Ο As a translation tool
Ο To generate images (DALL·E)
Ο To analyze data
Ο To write a scientific paper
Ο For data evaluation
Ο To search for references

**7. Could you imagine using ChatGPT to write a scientific paper, or have you already done so?**

Ο I could imagine using ChatGPT
Ο I have already used ChatGPT
Ο I do not think I will use the program

**8. In your opinion, should authors disclose if ChatGPT was used to write a paper?**

Ο Yes
Ο No
Ο I think this cannot be verified

**9. In your opinion, where does the main scientific contribution lie in the creation of a paper? (Multiple answers possible)**

Ο In the idea for the paper
Ο In data collection
Ο In data analysis
Ο In writing the paper

**10. Which of the following digital tools do you use when preparing a paper? (Multiple answers possible)**

Ο Search engines (Google etc.)
Ο Meta-databases (PubMed, Scopus etc.)
Ο Digital translation tools (DeepL, iTranslate etc.)
Ο Citation management tools (EndNote etc.)
Ο Tools for statistical analysis (SPSS, Excel etc.)
Ο Other

**Thank you for your participation!**
